# Risk factors of necrotizing enterocolitis among neonates admitted to the neonatal intensive care unit at the selected public hospitals in southern Ethiopia, 2023

**DOI:** 10.3389/fped.2024.1326765

**Published:** 2024-01-31

**Authors:** Mesfin Abebe, Mequanint Ayehu, Tsion Mulat Tebeje, Getnet Melaku

**Affiliations:** ^1^ Department of Midwifery, College of Medicine & Health Sciences, Dilla University, Dilla, Ethiopia; ^2^ Department of Nursing, College of Medicine & Health Sciences, Dilla University, Dilla, Ethiopia; ^3^ School of Public Health, College of Medicine & Health Sciences, Dilla University, Dilla, Ethiopia

**Keywords:** risk factors, necrotizing enterocolitis, neonates, Ethiopia, newborns

## Abstract

**Introduction:**

Necrotizing enterocolitis (NEC) is a serious intestinal condition characterized by ischemic necrosis of the intestinal mucosa, inflammation, and invasion by gas-forming organisms, posing a significant threat to neonatal health. Necrotizing enterocolitis remains a significant cause of neonatal morbidity and mortality, particularly in developing countries. Due to limited research conducted in Ethiopia and the study area, there is a lack of information regarding the risk factors associated with necrotizing enterocolitis. Therefore, the goal of this study is to fill the aforementioned gap.

**Objective:**

This study aims to identify the risk factors of necrotizing enterocolitis among neonates admitted to the neonatal intensive care unit (NICU) at selected general and referral hospitals in southern Ethiopia in the year 2023.

**Methods and materials:**

A facility-based unmatched case–control study was conducted. All neonates admitted to the NICU and diagnosed with necrotizing enterocolitis by the attending physician during the data collection period were considered as cases, whereas neonates admitted to the NICU but not diagnosed with necrotizing enterocolitis during the data collection period were considered as controls. Data were collected through face-to-face interviews and record reviews using the Kobo toolbox platform. The binary logistic regression method was used to determine the relationship between a dependent variable and independent variables. Finally, a *p*-value of < 0.05 was considered statistically significant.

**Results:**

This study included 111 cases and 332 controls. Normal BMI [AOR = 0.11, 95% CI: (0.02, 0.58)], history of khat chewing [AOR = 4.21, 95% CI: (1.96, 9.06)], term gestation [AOR = 0.06, 95% CI: (0.01, 0.18)], history of cigarette smoking [AOR = 2.86, 95% CI: (1.14, 7.14)], length of hospital stay [AOR = 3.3, 95% CI: (1.43, 7.67)], and premature rupture of membrane [AOR = 3.51, 95% CI: (1.77, 6.98)] were significantly associated with NEC.

**Conclusion:**

The study identified several risk factors for necrotizing enterocolitis, including body mass index, history of khat chewing, gestational age, history of cigarette smoking, length of hospital stays, and premature rupture of membrane. Therefore, healthcare providers should be aware of these risk factors to identify newborns at high risk and implement preventive measures.

## Introduction

Necrotizing enterocolitis (NEC) is a serious intestinal condition characterized by ischemic necrosis of the intestinal mucosa, as well as inflammation and enteric gas-forming organism invasion (1, 2). It is one of the most prevalent and fatal neonatal gastrointestinal crises (3, 4). NEC is a leading cause of death and morbidity in neonatal intensive care units, with a mortality rate of 15%–30% that rises with lower birth weight and preterm (5, 6). The worldwide incidence rate of necrotizing enterocolitis is 7% (7). In high-income countries, the incidence of necrotizing enterocolitis varied according to gestational age (GA), birth weight, and country (8). Evidence showed that in developed countries, the incidence rate of NEC among preterm and low birth weight babies was 2%–7% and 5%–22%, respectively (9). The studies conducted in the United States revealed that the incidence rate of NEC was 7%–10.7% (10, 11). Studies revealed that the prevalence rate of NEC was 4.87% in China (12), 25.4% in Addis Ababa (13), and 9.7% in Gurage (14). A study conducted in Indonesia found that 10% of full-term newborns had NEC (15).

Over 50% of infants diagnosed with NEC who require surgery die, and those with poor prognosis often experience neurological problems in their later years (11). The mortality rates for necrotizing enterocolitis affect approximately 25% of all infected newborns, and the survivors frequently experience long-term neurodevelopmental and/or gastrointestinal complications (10). It has a high medical cost for both families and society (16).

Prematurity, low birth weight, enteral feeding, blood transfusion, postnatal hypoxia, sepsis, meconium aspiration syndrome, and other factors have all been associated with NEC, according to numerous studies (16–19). Necrotizing enterocolitis has been associated with maternal factors such as chorioamnionitis, increased BMI, mode of delivery, placental abruption, preeclampsia, and smoking (8, 20).

Mortality and poor neurodevelopmental outcomes of infants with NEC remain high in resource-constrained regions (3). Despite advances in neonatal treatment, the overall mortality rate for necrotizing enterocolitis remains high, particularly in a newborn with advanced disease (8, 21). The Sustainable Development Goals (SDGs) include newborn health as a key component, and by 2030, countries are expected to have reduced neonatal mortality to at least 12 deaths per 1,000 live births (22). Necrotizing enterocolitis (NEC) continues to make a significant contribution to neonatal morbidity and mortality, especially in developing countries (18). Furthermore, a significant proportion of survivors experience neurodevelopmental delay, which lowers the quality of life for the patient and family and increases treatment costs (21). Necrotizing enterocolitis was diagnosed in approximately 4% of neonates admitted to the NICU, with a 46.2% fatality rate (23). Necrotizing enterocolitis was the leading cause of death in Ethiopia for preterm infants born at 28–31 weeks, 32–34 weeks, and 35–36 weeks of gestation, accounting for 22.2%, 55.6%, and 22.2% of deaths, respectively (24).

As a result, necrotizing enterocolitis is a serious condition that primarily affects premature babies and has a high mortality rate in Ethiopia. Preventive measures are required for a fatal disease that is very challenging to treat once it has been identified (25). However, to prevent necrotizing enterocolitis, we must first identify the risk factors. A few studies on the magnitude and associated factors of necrotizing enterocolitis have been conducted in Ethiopia, but all of them were conducted using a cross-sectional and retrospective approach. As a result, the current study differs in terms of study design and the inclusion of some variables that were not addressed in previous studies. Furthermore, there is a lack of data in the study setting and throughout the country that takes into account maternal lifestyle behaviors, pregnancy-related factors, and perinatal and neonatal factors associated with necrotizing enterocolitis, which is useful for designing contextual interventions.

## Methods and materials

### Study area, period, and design

The study was conducted from February 1, 2023 to August 31, 2023, among newborns admitted to the neonatal intensive care unit (NICU) in public hospitals of Hawasa and Dilla town, Southern Ethiopia. A facility-based unmatched case-control study was conducted.

### Population

The source population consisted of all neonates with index mothers who were admitted to the neonatal intensive care unit in Hawassa and Dilla town public hospitals. During the data collection period, all neonates with their index mothers were admitted to the neonatal intensive care unit in selected public hospitals in Hawassa and Dilla towns. These newborns met the inclusion criteria for both cases and controls, making them suitable for the study population.

## Eligibility criteria

### Inclusion criteria

All neonates who were admitted to the NICU at the selected public hospitals during the data collection period and neonates who were diagnosed with necrotizing enterocolitis (NEC) by the physician based on a standardized set of clinical and/or radiological criteria (modified bell stage criteria) or neonates who met the definition criteria of NEC and were admitted to the NICU at the selected public hospitals during the data collection period were considered as cases.

All neonates who were admitted to the NICU at the selected public hospitals during the data collection period and did not develop NEC during their stay in the NICU or neonates who did not meet the criteria for NEC and were admitted to NICU at the selected public hospitals during the data collection period were considered as controls.

### Exclusion criteria

**Cases:** Neonates who were initially diagnosed with NEC but subsequently recovered and had other health issues throughout the data collection period were excluded from the study. **Controls:** During the data collection period, neonates diagnosed with NEC and other neonatal diseases were excluded from the study. Neonates admitted to the NICU with major congenital anomalies or genetic disorders, neonates whose mothers were absent, deaf, or unable to communicate, and neonates with incomplete card information were excluded from the study as both cases and controls.

### Sample size determination

The sample size for an unmatched case–control study was calculated using the OpenEPI Info version 7 software and the double population proportion calculation, taking into account the variable of failure to breathe or be resuscitated from the previous study conducted in Addis Ababa (13), as it provided the largest sample size. The proportion of newborns who failed to breathe/were resuscitated among the control group is 21.3%, with a minimum detectable odds ratio (OR) of 2.1. This study assumed a precision of 5%, a power of 80%, and a case-to-control allocation ratio of 1:3. After accounting for a 10% non-response rate, the final sample size was 443 (111 cases and 332 controls).

### Sampling techniques and procedures

Three public hospitals [Adare General Hospital (AGH), Dilla University General Hospital (DUGH), and Hawassa University Comprehensive & Specialized Hospital (HUCSH)] were selected for this study. The sample size was allocated to the selected hospitals in proportion to their neonatal admission from the 6-month record of the previous year. Case–control incidence density sampling was used to select the study participants. Incidence density sampling involved selecting controls based on the diagnoses of the cases. For each diagnosed case, three controls were selected from the cohort who did not have the same diagnosis at that time. Due to the rarity of the case, each instance was included in the study until the required sample size was reached. Controls were selected on the day when the case was identified every *k* interval (*k* = 4), using a systematic sampling technique.

## Study variables

### Dependent variable

The dependent variable of the study was the necrotizing enterocolitis.

### Independent variables

The independent variables of the study were the **sociodemographic factors** (maternal age, sex of neonate, neonatal age, monthly income, maternal educational, and occupational status), **pregnancy-related factors** (parity, body mass index, preeclampsia, prolonged rupture of membrane, antenatal care (ANC) follow-up, maternal diabetes mellitus, maternal urinary infection, isoimmunization, placenta complication, prolonged labor, chorioamnionitis, use of steroid), **maternal lifestyle behavior** (drinking alcohol, smoking, khat chewing)**,** and **neonatal-related variables (**gestational age, birth weight, neonatal sepsis, neonatal asphyxia, multiple gestations, mode of delivery, length of hospital stay, meconium aspiration syndrome, enteral feeding**).**

### Operational and term definitions

**Necrotizing enterocolitis** is a surgical disorder of the gastrointestinal tract that manifests clinically as abdominal distention, abdominal wall erythema, feeding intolerance, vomiting, and blood in the stool in neonates. Radiologically, it is identified by the presence of dilated bowel loops, bowel wall edema, pneumatosis intestinalis, portal venous gas, and pneumoperitoneum (14, 26).

### Criteria for diagnosing NEC

Necrotizing enterocolitis is commonly diagnosed by evaluating a combination of clinical signs and radiological findings, and it is also classified according to modified Bell's staging system (27).

### Data collection tools and procedures

After reviewing previous related literature, the investigators prepared a structured interview-administered questionnaire and checklist to review medical records. The medical record checklist includes maternal sociodemographic, pregnancy-related, and perinatal and neonatal variables. An exit face-to-face interview and record review were used to collect data. Six midwives with BSc degrees were employed to collect the data, while two supervisors with MSc degrees were recruited for supervisory duties. The researchers provided theoretical and practical training to data collectors and supervisors regarding data collection instruments, interviewing methodologies, information confidentiality, and the purpose and significance of the study. The data collector's Android phone was equipped with the Kobo Collect version 3.1 program, and the blank form was obtained from the Kobo toolbox server.

### Data quality management

A language expert translated the questionnaire into the local language to ensure data quality. The data collectors and supervisors were trained for 2 days to become familiar with different types of data, tools, and data collection procedures and objectives, as well as a 1 day training session on Kobo Collect practice. The questionnaire was pre-tested 3 weeks prior to the actual data collection at Yirgalem General Hospital, which shares a similar status with the study area. To ensure data quality, the supervisors reviewed the completed questionnaires for critical items before uploading them from the Android mobile phone to the Kobo toolbox server. All data were collected on-site using Android mobile devices and transmitted to the Kobo server on a weekly basis using Kobo collect version 3.1. The principal investigator verified the files submitted by each data collector regularly for consistency and completeness. Finally, suitable data coding and categorization were performed to verify data quality.

### Data processing and analysis

The data from the Kobo server were downloaded as an Excel file and imported into StataSE V.17 software for cleaning, coding, confirming completeness and accuracy, and further analysis. The outcome variable was divided into two categories: cases and controls (1 = cases, 0 = controls). A descriptive analysis was performed to describe the pertinent characteristics of the study participants. A binary logistic regression was used to examine the relationship between each independent variable and the outcome variable. The Hosmer and Lemeshow test was used to determine the goodness of fit. To control for any possible confounding variables, the variables from the bivariate analysis with a 95% confidence interval and a *P*-value < 0.25 were included in the multivariable logistic regression analysis. Furthermore, the factors that were found to be significant in previous studies and relevant from a contextual perspective were included in the final model, regardless of whether they satisfied the aforementioned criteria. The independent effect was found using the forward logistic regression model. Colinearity diagnostic statistics were used to screen for multicollinearity using variance inflation factors and a tolerance test. *P*-values less than 0.05 were considered statistically significant, as were adjusted odds ratios with a 95% confidence interval. Finally, data were presented in the form of tables and texts.

## Results

### Sociodemographic, economic, and lifestyle behavioral characteristics of the study participants

This study included 111 cases and 332 controls, with a 100% response rate. The mean age of the mothers was 28.33 years, with a standard deviation of 4.66 years. The majority of the case mothers (61, 54.95%) and control mothers (224, 67.47%) were between the ages of 25 and 34 years. In terms of residence, 43 (38.74%) of the case mothers and 123 (37.05%) of the control mothers lived in rural areas. Approximately 87 (78.38%) of the cases and 287 (86.45%) of controls were married. A total of 57 (51.35%) cases and 149 (44.88%) controls had primary education levels in terms of educational status. Of the respondents, 10 (9.01%) cases and 10 (3.01%) controls had a higher body mass index. During their pregnancy, 10 (10.01%) cases and 44 (13.25%) controls had a history of alcohol consumption. Regarding the sex of newborns, 85 cases (76.58%) and 148 controls (44.58%) were male (Table 1).

**Table 1:** Sociodemographic, economic, and lifestyle behavioral characteristics of the study participants, southern Ethiopia, 2023 (*N* = 443).

Variables	Cases (*n* = 111)	Controls (*n* = 332)
Maternal age (in years)		
15–24	36 (32..43)	49 (14.76)
25–34	61 (54.95)	224 (67.47)
≥35	14 (12.61)	59 (17.77)
Residence		
Urban	68 (62.26)	209 (62.95)
Rural	43 (38.74)	123 (37.05)
Marital status		
Single	12 (10.81)	17 (5.12)
Married	87 (78.38)	287 (86.45)
Divorced	6 (5.41)	8 (2.41)
Separated	6 (5.41)	20 (6.02)
Educational status		
No formal education	19 (17.12)	78 (23.49)
Primary	57 (51.35)	149 (44.88)
Secondary	25 (22.52)	95 (28.61)
College and above	10 (9.01)	10 (3.01)
Body mass index		
<18.5 kg/m^2^ (low)	19 (17.12)	64 (19.28)
18.5–24.9 kg/m^2^ (normal)	82 (73.87)	258 (77.71)
≥25 kg/m^2^ (high)	10 (9.01)	10 (3.01)
Occupational status		
Government employee	15 (13.51)	57 (17.17)
Merchant	42 (37.84)	74 (22.29)
Farmer	20 (18.02)	53 (15.96)
Housewife	23 (20.72)	117 (35.24)
Other^a^	11 (9.91)	31 (9.34)
Alcohol use history during current pregnancy		
Yes	10 (9.01)	44 (13.25)
No	101 (90.99)	288 (86.75)
Khat chewing history during current pregnancy		
Yes	42 (37.84)	91 (27.41)
No	69 (62.16)	241 (72.59)
Cigarette smoking history during current pregnancy		
Yes	25 (22.52)	39 (11.75)
No	86 (77.48)	293 (88.25)
Sex of neonates		
Male	85 (76.58)	148 (44.58)
Female	26 (23.42)	184 (55.42)
Neonatal age		
<7 days	41 (36.94)	193(58.13)
≥7 days	70(63.06)	139 (41.87)

^a^ daily labor, student, unemployed, and unspecified.

### Pregnancy-related characteristics of the study participants

Out of the participants in the study, 94 (84.68%) of the cases and 241 (72.59%) of controls were identified as multiparous mothers. During their pregnancy, approximately 83 (74.77%) of the cases and 224 (67.47%) of controls received antenatal care. Gestational hypertension was found in 11 (9.91%) of the cases and 29 (8.73%) of controls. Eighteen (16.22%) of the case mothers and 140 (42.17%) of controls had their labors prolonged. Premature rupture of membrane (PROM) was found in 84 (75.68%) of the case mothers and 108 (32.53%) of control mothers (Table 2).

**Table 2:** Pregnancy-related characteristics of the study participants, southern Ethiopia, 2023 (*N* = 443).

Variables	Cases (*n* = 111)	Controls (*n* = 332)
Parity		
Primiparous	17 (15.32)	91 (27.41)
Multiparous	94 (84.68)	241 (72.59)
History of ANC follow-up		
Yes	83 (74.77)	224 (67.47)
No	28 (25.23)	108 (32.53)
History of gestational hypertension		
Yes	11 (9.91)	29 (8.73)
No	100 (90.09)	303 (91.27)
History of diabetes mellitus		
Yes	5 (4.50)	48 (14.46)
No	106 (95.50)	284 (85.54)
History of UTI		
Yes	66 (59.46)	110 (33.13)
No	45 (40.54)	222 (66.87)
RH Isoimmunization history		
Yes	9 (8.11)	75 (22.59)
No	102 (91.89)	257 (77.41)
Placenta complication history during the current pregnancy		
Yes	13 (11.71)	79 (23.80)
No	98 (88.29)	253 (76.20)
History of chorioamnionitis		
Yes	46 (41.44)	104 (31.33)
No	65 (58.56)	228 (68.67)
Steroid drug usage history during pregnancy		
Yes	41 (36.94)	42 (12.65)
No	70 (63.06)	290 (87.35)
Number of current pregnancy		
Single	85 (76.58)	285 (85.84)
Twin	26 (23.42)	47 (14.16)
Labor prolonged		
Yes	18 (16.22)	140 (42.17)
No	93 (83.78)	192 (57.83)
PROM		
Yes	84 (75.68)	108(32.53)
No	27(24.32)	224(67.47)

### Perinatal and neonatal-related characteristics of the study participants

Among the study participants, 93 (83.78%) of the cases and 125 (37.65%) of control newborns were delivered at a preterm gestational age, and 76 (68.47%) of the cases and 88 (26.51%) of control newborns were delivered with less than 2,500 g birth weight. It was reported that 23 of the cases (20.72%) and 137 of controls (41.27%) had a history of neonatal sepsis. Regarding the mode of delivery, 89 of the cases (80.18%) and 156 of controls (46.99%) had spontaneous vaginal deliveries. Trophic feeding was given to approximately 66 (59.46%) of the cases and 220 (66.27%) of controls. Regarding the type of initial milk feeding, breast milk was provided first to 71 (63.96%) of the cases and 230 (69.28%) of controls (Table 3).

**Table 3:** Perinatal and neonatal-related characteristics of the study participants, southern Ethiopia, 2022 (*N* = 443).

Variables	Cases (*n* = 111)	Controls (*n* = 332)
Gestational age at the time of delivery		
Term gestation	11 (9.91)	197 (59.34)
Preterm gestation	93 (83.78)	125 (37.65)
Post term gestation	7 (6.31)	10 (3.01)
Birth weight		
<2.5 kg	76 (68.47)	88 (26.51)
2.5–3.9 kg	26 (23.42)	224 (67.47)
≤4.kg	9 (8.11)	20 (6.02)
History of neonatal sepsis		
Yes	23 (20.72)	137 (41.27)
No	88 (79.28)	195 (58.73)
History of birth asphyxia		
Yes	16 (14.41)	60 (18.07)
No	95 (85.59)	272 (81.93)
Mode of delivery		
SVD	89 (80.18)	156 (46.99)
Instrumental	10 (9.01)	85 (25.60)
Cesarean Section	12 (10.81)	91 (27.41)
Length of hospital stay		
≤7 days	63 (56.76)	203 (61.14)
>7 days	48 (43.24)	129 (38.86)
History of meconium aspiration syndrome		
Yes	14 (12.61)	53 (15.96)
No	97 (87.39)	279 (84.04)
Trophic feeding received		
Yes	66 (59.46)	220 (66.27)
No	45 (40.54)	112 (33.73)
Type of milk feeding		
Breast milk	71 (63.96)	230 (69.28)
Formula milk	33 (29.73)	79 (23.80)
Mixed	7(6.31)	23(6.93)

### Risk factors of necrotizing enterocolitis

To determine the risk factors of NEC, the variables with a *p*-value of less than 0.25 in the bivariate analysis, significant associations in prior studies, and biological plausibility were considered for the multivariable model in this study. In the binary logistic regression analysis, maternal age, history of khat chewing, antenatal care follow-up, gestational age of newborn, birth weight, gestational hypertension, history of cigarette smoking, length of hospital stay, and PROM were identified as candidate variables for the final multivariable logistics regression analysis model. Finally, body mass index, khat chewing history, gestational age, history of cigarette smoking, length of hospital stay, and PROM were all found to be significantly associated with necrotizing enterocolitis in the multivariable analysis.

Neonates born to mothers with a normal body mass index had an 89% lower likelihood of developing NEC compared with neonates with mothers who had their body mass index of ≥ 25 kg/m^2^ [AOR = 0.11, 95% CI: (0.02, 0.58)]. Neonates whose mothers chewed khat during their pregnancy were 4.21 times more likely to develop necrotizing enterocolitis than neonates whose mothers did not chew khat during their pregnancy [AOR = 4.21, 95% CI: (1.96, 9.06)]. Newborns who had delivered at a term gestation were 94% less likely to have necrotizing enterocolitis than those newborns delivered at a preterm gestation [AOR = 0.06, 95% CI: (0.01, 0.18)].

Neonates whose mothers had a history of cigarette smoking were 2.86 times more likely to have necrotizing enterocolitis than neonates whose mothers without a history of cigarette smoking [AOR = 2.86, 95% CI: (1.14, 7.14)]. The odds of having necrotizing enterocolitis were 3.3 times more likely in neonates who stay at the hospital for more than 7 days than newborns who stay at the hospital ≤ 7 days [AOR = 3.3, 95% CI: (1.43, 7.67)]. Neonates born with PROM were 3.51 times more likely to have necrotizing enterocolitis than neonates without PROM [AOR = 3.51, 95% CI: (1.77, 6.98)] (Table 4).

**Table 4:** Bivariate and multivariable logistic regression analysis of factors associated with necrotizing enterocolitis among the study participants, southern Ethiopia, 2023 (*N* = 443)*.*

Variables	Necrotizing enterocolitis		COR (95% CI)	AOR (95% CI)	*P*-value
	Cases	Controls			
Body mass index					
<18.5 kg/m^2^	19 (17.12%)	64 (19.28%)	0.29 (0.10, 0.81)	0.26 (0.04, 1.55)	0.14
18.5–24.9 kg/m^2^	82 (73.87%)	258 (77.71%)	0.31 (0.12, 0.79)*	0.11 (0.02, 0.58)**	**0.009**
≥25 kg/m^2^	10 (9.01%)	10 (3.01%)	1	1	
Khat chewing history during the current pregnancy					
Yes	42 (37.84%)	91 (27.41%)	1.61 (1.02, 2.53)	4.21 (1.96, 9.06)**	**<0.001**
No	69 (62.16%)	241 (72.59%)	1	1	
ANC follow-up during pregnancy					
Yes	83 (74.77%)	224 (67.47%)	1	1	
No	28 (25.23%)	108 (32.53%)	0.69 (0.43, 1.13)	1.72 (0.87, 3.38)	0.11
Gestational age of newborn					
Term gestation	11 (9.91%)	197 (59.34%)	0.07 (0.03, 0.14)*	0.06 (0.01, 0.18)**	**<0.001**
Preterm gestation	93 (83.78%)	125 (37.65%)	1	1	
Postterm	7 (6.31%)	10 (3.01%)	0.94 (0.34, 2.56)	0.54 (0.07, 3.80)	0.53
Birth weight					
<2.5 kg	76 (68.47%)	88 (26.51%)	1	1	
2.5–3.9 kg	26 (23.42%)	224 (67.47%)	0.13 (0.08, 0.22)*	0.94 (0.42, 2.09)	0.89
≥4 kg	9 (8.11%)	20 (6.02%)	0.52 (0.22, 1.21)	2.01 (0.28, 14.21)	0.48
Presence of gestational hypertension					
Yes	11 (9.91%)	29 (8.73%)	1	1	
No	100 (90.09%)	303 (91.27%)	0.87 (0.41, 1.80)	1.64 (0.31, 8.15)	0.55
History of cigarette smoking					
Yes	25 (22.52%)	39 (11.75%)	2.18 (1.25, 3.81)*	2.86 (1.14, 7.14)**	**0.02**
No	86 (77.48%)	293 (88.25%)	1	1	
Length of hospital stay					
≤7 day	63 (56.76%)	203 (61.14%)	1	1	
>7 day	48 (43.24%)	129 (38.86%)	1.19 (0.77, 1.85)	3.33 (1.43, 7.67)**	**0.004**
PROM					
Yes	84 (75.68%)	108 (32.53%)	6.45 (3.95, 10.53)*	3.51(1.77, 6.98)**	**<0.001**
No	27(24.32%)	224(67.47%)	1	1	

* statistically significant at bivariate analysis.

** statistically significant at multivariable analysis with *P*-value < 0.05.

## Discussion

Necrotizing enterocolitis is one of the most common gastrointestinal emergencies in newborns (28). It is a potentially fatal gastrointestinal disease frequently observed in NICUs worldwide. NEC causes ischemia and necrosis in some areas of the infant's gastrointestinal system and has a reported mortality rate of up to 25% (29).

Although the exact cause of NEC is still unknown, several risk factors have been identified. Thus, determining the risk factors associated with necrotizing enterocolitis is also beneficial in developing guidelines that aim to prevent contributing factors, lower the burden on the healthcare system, and promote community health. This study indicated that necrotizing enterocolitis was significantly associated with body mass index, history of khat chewing, gestational age, history of cigarette smoking, length of hospital stays, and premature rupture of membrane.

Maternal BMI has been found as a possible risk factor for necrotizing enterocolitis. This study found that neonates born to mothers with a normal BMI were 89% less likely to have NEC than neonates born to mothers with a higher BMI. The finding of this study is consistent with the previous study conducted in Canada (8). The findings of this study contradict those of a Swedish study (19). The mechanism by which maternal BMI increases the risk of necrotizing enterocolitis is not yet understood. The possible reason may be that obesity in mothers may change the composition of breast milk, which may affect the newborn's gut microbiota and increase the risk of necrotizing enterocolitis. Inflammation and oxidative stress, which are both recognized to be increased in obese individuals, are other plausible causes. These factors can increase the risk of NEC by inducing intestinal inflammation and damage. However, further research is needed to fully understand these relationships.

The risk of necrotizing enterocolitis was 4.21 times higher in mothers who chewed khat while pregnant. This could be explained by the fact that chewing khat during pregnancy may lead to a variety of obstetric complications, including preterm birth, low birth weight, and premature membrane rupture (30–33), secondary to which the newborn may increase the risk of necrotizing enterocolitis, according to the critical review results conducted by the WHO Expert Committee on Drug Dependence (ECDD) (34) and other studies. Another explanation might be the vasoconstrictive effects of the component khat cathinone, which can lower blood flow to the digestive system and increase the risk of NEC. NEC is a severe gastrointestinal disease that is believed to be influenced by the gut microbiota (35). Therefore, it is plausible to suggest that changes in the oral microbiota induced by khat chewing could potentially influence the gut microbiota, consequently affecting the risk of developing NEC (36). In conclusion, while khat chewing has been associated with modifications in the periodontal microbiota as a risk factor for NEC, it is complex and likely involves multiple factors. Further research is needed to fully understand these relationships.

The gestational age was found to be a risk factor for NEC in this study. Necrotizing enterocolitis was 94% less likely in neonates delivered at full-term gestation than in newborns delivered during preterm gestation. This finding is supported by the studies conducted in Ethiopia (13, 37), China (38), Korea (39), the Netherlands (40), Taiwan (41), Indonesia (42), and Canada (8). Because the intestines of a preterm newborn are not fully matured, they are more susceptible to infections and inflammation. The most common cause of NEC is prematurity. This can be explained by ischemia mucosal damage in the underdeveloped gut of the preterm newborns.

Neonates whose mothers had a history of cigarette smoking were 2.86 times more likely to have necrotizing enterocolitis than neonates whose mothers did not have a history of cigarette smoking. Other studies conducted in Louisville (43) and Canada (8) also revealed that smoking cigarettes during their pregnancy was significantly associated with necrotizing enterocolitis. Pieces of evidence revealed that smoking during pregnancy is a well-established risk factor that increases the likelihood of adverse pregnancy outcomes, including preterm birth and low birth weight (44, 45). These factors, in turn, increase the risk of developing NEC. Premature newborns have underdeveloped bowels, rendering them more susceptible to developing NEC compared with full-term babies. This finding is inconsistent with a study conducted in Sweden (19).

The odds of having necrotizing enterocolitis were 3.3 times more likely in neonates who stay at the hospital for more than 7 days than in newborns who stay at the hospital for ≤ 7 days. A similar finding was also reported in Ethiopia (14) and Iran (46). This could be related to the possibility of developing nosocomial infection while staying in the hospital for an extended period.

The finding of this study also found that premature rupture of the membranes could raise the likelihood of developing NEC. Neonates born with PROM were 3.51 times more likely to have necrotizing enterocolitis than neonates without PROM. This finding is consistent with the studies conducted in Ethiopia (37), China (38), Atlanta (47), and the Netherlands (40). The possible explanation could be that the fetus is more exposed to the bacteria in the vaginal canal as a result of the premature rupture of the membrane. The gastrointestinal tract may then become infected and inflamed as a result of this bacterial colonization. This may make the newborn more vulnerable to necrotizing enterocolitis following birth. In contrast, studies conducted in Sweden and China found no statistically significant association between premature membrane rupture and necrotizing enterocolitis (19, 48). This discrepancy may be caused by differences in the study design and sample size. Our study has some limitations that need to be acknowledged. First, we did not include variables such as gut dysbiosis and perinatal antibiotic exposure, which may have risk factors for NEC. Future studies should consider these variables and their potential interactions with other risk factors. Second, our study was conducted in a hospital setting and limited to a single region in Ethiopia. Therefore, our findings may not be generalizable to other populations or settings. Further research is needed to validate our results and explore the epidemiology and risk factors of NEC in different contexts.

## Conclusion and recommendation

This study identified several risk factors for necrotizing enterocolitis, including body mass index, history of khat chewing, gestational age, history of cigarette smoking, length of hospital stays, and premature rupture of the membrane. Therefore, healthcare providers should be aware of these risk factors to identify high-risk newborns and implement preventive measures.

## Data Availability

To protect participant confidentiality, the datasets generated and/or analyzed for this study are not publicly available. However, they are available from the corresponding author upon reasonable request.
